# Integrating Gender-Based Violence Screening and Support into the Research Clinic Setting: Experiences from an HIV Prevention Open-Label Extension Trial in Sub-Saharan Africa

**DOI:** 10.1007/s10461-022-03864-6

**Published:** 2022-09-30

**Authors:** Morgan Garcia, Sarah T. Roberts, Ashley J. Mayo, Rachel Scheckter, Leila E. Mansoor, Thesla Palanee-Phillips, Krishnaveni Reddy, Yuthika Naidoo, Carolyne Agwau Akello, Zakir Gaffoor, Samantha Siva, Chenai Rushwaya, Kudzai Hlahla, Jane Jambaya, Rujeko Makoni, Evans Kachale, Margret Ndovie, Jabulisile Zuma, Elizabeth T. Montgomery, the MTN-025/HOPE study team

**Affiliations:** 1 Global Health Population and Nutrition, FHI 360, Durham, NC USA; 2 Women’s Global Health Imperative (WGHI) RTI International, Berkeley, CA USA; 3 Centre for the AIDS Programme of Research in South Africa (CAPRISA), University of KwaZulu-Natal, Durban, South Africa; 4 Faculty of Health Sciences, Wits Reproductive Health and HIV Institute (Wits RHI), University of the Witwatersrand, Johannesburg, South Africa; 5 University of Washington School of Public Health, Seattle, WA USA; 6 Makerere University-Johns Hopkins University (MU-JHU) Research Collaboration, Kampala, Uganda; 7 HIV Prevention Research Unit (HPRU), South African Medical Research Council (SAMRC), Durban, South Africa; 8 University of Zimbabwe Clinical Trials Research Centre, Harare, Zimbabwe; 9 College of Medicine – Johns Hopkins Bloomberg School of Public Health, Blantyre, Malawi; 10 University of North Carolina Project, Lilongwe, Malawi; 11 Desmond Tutu HIV Foundation (DTHF) - Emavundleni Clinical Research Site, Cape Town, South Africa; 12 Global Health Population and Nutrition, FHI 360, 359 Blackwell St, Ste 200, Durham, NC 27701 USA

**Keywords:** HIV prevention, Gender-based violence response, Dapivirine vaginal ring, Referral networks, Vicarious trauma

## Abstract

HIV and gender-based violence (GBV) are syndemic in sub-Saharan Africa and provision of support for participants who disclose GBV constitutes part of comprehensive care. Consequently, a process was undertaken to develop, implement, and evaluate standard operating procedures (SOPs) in MTN-025/HOPE, a study of the dapivirine vaginal ring for HIV prevention. The SOP was developed using needs assessment surveys in addition to World Health Organization (WHO) guidelines and other literature. Sites tailored and implemented the SOP through HOPE implementation. At study end, staff reported increased training 32/35 (91.43%); improved confidence (18/26; 69.23%); and improved vicarious trauma prevention onsite (17/28; 60.71%). Leadership reported increased staff competence in GBV response. Obstacles included limited referral organizations and time for follow-up, continued training needs, and cultural norms. Development and implementation of an SOP is a feasible strategy to build a GBV response to improve health systems and support sustained effective use of HIV prevention products.

**Supplementary Information:**

The online version contains supplementary material available at 10.1007/s10461-022-03864-6.

## Introduction

Research into antiretroviral-based HIV pre-exposure prophylaxis (PrEP) methods has expanded prevention possibilities, introducing a variety of user-initiated methods—and efforts to support effective use of these methods—that are key to reducing HIV incidence among women, who have been disproportionately impacted by the pandemic [1]. In March 2021, the updated World Health Organization (WHO) guidelines included one of these methods, the monthly dapivirine vaginal ring (“the ring”), as an additional option for cisgender women, laying the foundation for the introduction of the world’s first long-acting HIV prevention product [2]. Other methods, including injectable cabotegravir, as well as multipurpose prevention technologies that prevent both pregnancy and HIV or other sexually transmitted infections, are also in various stages of development and regulatory review [3–5]. There is a growing body of evidence indicating that gender-based violence (GBV), especially intimate partner violence (IPV), significantly impacts women’s willingness and ability to successfully utilize PrEP [6, 7]. Socio-economic and structural factors contribute to HIV and GBV incidence, and recent meta-analyses estimate that GBV prevalence among women in sub-Saharan Africa is approximately 44.4%, with women and girls making up 63% of new HIV diagnoses in the region in 2020 [8–14].

Due to the syndemic relationship between HIV and GBV, multiple international donors have recently recognized that expanded and improved GBV response and prevention is a key component of comprehensive HIV services [15]. Similarly, improvement of these services at research sites where HIV prevention methods are under investigation offers the opportunity to provide more comprehensive care to participants, and potentially to improve adherence to novel PrEP methods by removing barriers to adherence—such as providing support to leave a violent relationship—or supporting participant skills to navigate PrEP use in the context of a violent relationship through provision of tools for safe disclosure or discreet use, although further research is needed in this area [16, 17]. However, limited guidance exists for HIV service providers and research sites seeking to integrate GBV response into their practice.

Multi-disciplinary groups, such as sexual assault response teams, have long been used to enhance the quality, accessibility, and predictability of services for violence survivors [18, 19]. These multi-disciplinary groups bring together disparate responders and service providers, such as representatives from health care facilities, nursing organizations, mental health service points, law enforcement, protection services, and victims’ advocacy groups to develop standard collaborative processes and working agreements, combining diverse expertise in the service of survivor interests. Such teams are common across the United States and similar collaboration is recommended by the Inter-Agency Standing Committee Taskforce on Gender in Humanitarian Assistance and the Inter-Agency Working Group for Reproductive Health in Crises, and the WHO [20–22]. In addition, programming and research efforts related to violence response can present a risk of vicarious trauma to researchers and implementors, particularly for those who have also experienced violence [23, 24].

In MTN-025/HOPE, an open-label extension trial of the ring among women in sub-Saharan Africa, it was thought that open conversation and disclosure fostered by study-specific counseling, coupled with routine questions examining experiences of violence in a context of high rates of GBV within communities where the study took place and among likely participants, would increase the likelihood of GBV disclosure during study participation [25–27]. To better meet the needs of study participants and staff, efforts were made to improve site teams’ GBV response capacity by incorporating lessons learned from multidisciplinary GBV response practice and vicarious trauma prevention into existing site structures through the creation and implementation of a template standard operating procedure (SOP) for GBV response [28]. This paper describes the SOP development and implementation process, as well as outcomes of a process evaluation, to broaden the knowledge base regarding support for GBV survivors.

## Methods

### Study Population and Design

The HIV Open-label Prevention Extension (HOPE) study (NCT02858037) was a phase 3B trial of the ring for HIV prevention, which enrolled 1456 former participants of the MTN-020/ASPIRE (NCT01617096) phase 3 trial at 14 clinical research sites in Malawi, South Africa, Uganda, and Zimbabwe [29]. HOPE participants were consented per local institutional review board-approved processes prior to enrollment and followed for approximately 12 months. Participants chose whether to use the ring throughout study participation and were supported to make this choice using a client-centered counseling approach [30]. HOPE results reinforced the ring’s favorable safety profile and illustrated willingness to use the ring, with more than 73% of participants choosing the ring throughout follow-up, and more than 89% of returned rings indicating at least some use [28]. Additional details regarding study design, recruitment, and results have been published previously [28].

### SOP Design and Implementation

To ensure that site teams were prepared to identify and respond to participants who reported GBV, the study management team conducted two needs assessment surveys—one among site staff and one among site leadership—and developed and implemented a template GBV Response SOP based on identified needs, best practices in multidisciplinary GBV response, and international guidelines regarding provision of services for individuals experiencing GBV [18, 22]. Because this activity was conducted to improve study implementation and efforts for participant support and to reduce the impact of compassion fatigue among staff, and not part of study data collection, participant needs were identified based on analyses of social harms, GBV, and male partner influence collected during the ASPIRE study, from which HOPE participants were drawn [25–27, 31, 32]. In addition, two process assessment surveys were conducted after SOP implementation, one each with site staff and leadership, to evaluate the SOP implementation experience. Thirteen of the 14 HOPE sites implemented the SOP; the last site was participating in a separate study of an integrated PrEP and IPV counseling intervention called the Community Health clinic model for Agency in Relationships and Safer Microbicide Adherence (CHARISMA), following separate study-specific procedures and SOPs [33, 34]. The CHARISMA site was included in the needs assessment but did not complete the process evaluation.

#### Needs Assessment Surveys

To understand existing resources and needs at HOPE sites, and inform SOP development, site team members were requested via email to complete two needs assessment surveys in Qualtrics. Surveys were anonymous and contained 10–15 multiple choice, LIKERT scale, and open-ended questions, depending on respondent experience and resources to report. Sites were requested to assign 3–4 individuals to complete the survey: 1 leadership representative (a total of 14 representatives) and 2–3 staff per site including counselors, nurses, interviewers, and community educators who most commonly interacted directly with participants (about 28–42 total staff). This sampling was predicted to be broadly representative as each site had 1–3 individuals in study leadership and, while sites likely had more than 15 staff assigned to the study, only about 5–10 of these staff had regular in-depth interactions with HOPE participants. The survey addressed: GBV training, referral resources and staff requirements for understanding these resources, confidence in responding to GBV, standardized procedures for GBV response, and feelings of support within their teams. The leadership survey, completed by site investigators of record (IoRs), study coordinators, sub-investigators, and site leaders, focused on GBV training (including trainers and follow-up requirements), referral partners, standardized GBV response procedures, and mechanisms for receiving participant feedback on referral organizations.

#### SOP Template Development

The HOPE GBV Response SOP template provided a framework for sites to increase basic staff training on the dynamics of GBV and the provision of first-line, trauma-informed care, expand and strengthen referral networks through investigation and relationship development, improve staff support to prevent vicarious trauma by establishing regular debrief sessions and psychological support options, encourage participant sensitization on GBV, and build a shared understanding of staff roles and site approaches to identifying and responding to GBV among participants [35].

Key resources, such as the WHO Clinical and Policy Guidelines for Responding to Intimate Partner Violence and Sexual Violence Against Women and United Nations Population Fund Multi-Sectoral Response to GBV Guide, were cross-referenced with results of the needs assessment surveys to identify key components for the SOP template [22, 36]. This review, coupled with gaps identified via the surveys, led to the inclusion of key definitions, training requirements, and procedures for GBV identification, provision of first-line support, referral, and follow-up. In addition, vicarious trauma prevention trainings, such as those provided by the Headington Institute, were reviewed for tools to facilitate a supportive culture to enable site teams to continue providing first-line and long-term follow-up to participants [37].

#### SOP Adaptation

Study management supported sites to adapt the SOP template to reflect local resources, site structure, and staff roles. Site teams reviewed and revised lists of referral organizations based on existing needs and template SOP recommendations and conducted outreach when needed. Site leadership engaged local organizations to deliver staff trainings and support participant GBV sensitization via on-site visits and attendance at participant engagement activities (PEAs), which also provided an opportunity for participants to become familiar with referral organizations [38]. Where local resources were limited, study management supported new connections via regional networks, linking teams to individuals and organizations working to improve human rights in study countries. Study management also helped sites make confidential counseling available to staff, assisting in the identification of support organizations, encouraging investment in psychology sessions, and problem solving so that staff could access existing support within their sites. Multiple sites also engaged professional psychologists to improve processes and resources for staff. Site-specific SOPs adapted from the template were finalized with approval from the management team and study site leadership.

#### SOP Process Evaluation Surveys

At study end, a second set of anonymous surveys was conducted—one each for staff and leadership—comprising 19–25 multiple choice, LIKERT scale, and open-ended questions, depending on referral resources and experience with management team support, to assess SOP adaptation and implementation. Although these surveys were administered to the same categories of staff members and leaders as the first surveys, with the same instructions for participant selection, respondents differed due to staff turnover and availability. Because the surveys were anonymous and respondents were not identified, it is not known how many respondents completed both surveys or the overlap in responses. Topics addressed included trainings, on-site support for staff, confidence in responding to GBV, and standardized site processes.

### Analysis

All four surveys were summarized from Qualtrics data using descriptive statistics and synthesis of qualitative responses to open-ended questions. Response proportions were calculated with number of respondents selecting a given response representing the numerator, while total respondents to the question served as the denominator. Comparisons were made between responses on the needs and process evaluation surveys and proportion of respondents selecting a given response to capture changes reported by site staff and leadership. Illustrative quotes highlighting key themes are presented in the Results section. Site PEA reports were reviewed for mention of referral partner engagement and inclusion of GBV-related topics with instances of referral partner engagement summarized.

## Results

### Needs Assessment Surveys

A total of 34 site staff (1–5 per site from 13 of 14 sites) and 17 site leadership representatives (1–3 respondents each from 11 of 14 sites) completed the baseline needs assessment.

Of staff, 38% (13/34) had received some previous training in GBV-related topics as part of their work at the research site. Among 26 staff with prior experience supporting participants reporting violence, 21 (80%) had reached out to site leadership for support, 13 (50%) reached out to fellow staff and 6 (23%) contacted outside organizations. Staff were split on standardized responses to GBV: 12 of 26 (46%) reported a standard site-wide process, an additional 12 of 26 (46%) reported following study-specific processes, and 2 of 26 (8%) reported that there was no standard process for GBV response. Nearly all respondents (92%; 23/25) reported that there was at least one organization in their area that offered services to survivors of GBV via written referral (78%; 18/23) or walk-in (48%; 11/23). Only 35% (8/23) of respondents reported using site staff accompaniment during referrals. When asked what they did well when responding to GBV, most respondents mentioned listening (36%; 9/25) or supportive counseling (28%; 7/25).

Eight (47%) leadership respondents indicated that staff received prior training on GBV prevention, support, and response, with 71% (10/14) indicating that there were no SOPs or other standard resources on-site for responding to participants who disclosed GBV. While nearly all respondents (93%; 13/14) indicated that their site provided referrals for GBV services, referrals reportedly occurred yearly (8%; 1/13) or less (92%; 12/13). Of sites with known referral organizations, 54% (7/13) collected follow-up information on participant contacts with at least some of these organizations. Like staff, site leadership identified counseling as a strength in responding to GBV (58%; 7/12). Although site leadership indicated few referrals, 58% (7/12) also cited access to referral options as a strength.

Finally, respondents were asked what was needed to improve GBV response at their site. Responses to this open-ended question are outlined thematically in Table 1.

**Table 1:** Site-reported needs for improving GBV response

Response category	Site leadership (12*)	Site staff (24*)	Total
	% (N)	% (N)	% (N)
GBV training	33% (4)	54% (13)	47% (17)
Improved referral networks	17% (2)	21% (5)	19% (7)
Further counseling for participants	33% (4)	4% (1)	14% (5)
Life skills training for participants	0	8% (2)	6% (2)
Staff debriefing	0	8% (2)	6% (2)
GBV response SOP	0	8% (2)	65 (2)
Material support for participants	17% (2)	0	6% (2)
Medical treatment for participants	17% (2)	0	6% (2)
Other	8% (1)	13% (3)	11% (4)

*Five site leadership respondents and 10 site staff respondents skipped this question

### SOP Implementation

Because the SOP process was conducted to improve study services, all participants at the 13 sites received care per the SOP. Per standard study practice, all staff cadres were trained on their site-specific SOP. Sites then employed multiple strategies to ensure sufficient GBV training as required by the SOP, including inviting community advisory board members, consultants, and external service organizations to provide trainings, or utilizing online trainings provided by groups such as MOSAIC Training, Service, and Healing Centre and the United Nations High Commissioner for Refugees. Trainings were recommended for all cadres, from outreach workers to clinicians, to foster a supportive research environment. Per SOP recommendations, some sites also initiated multi-disciplinary GBV response teams including staff cadres who received GBV training. Training organizations were added to site referral directories, with staff providing accompanied referrals when accepted by participants.

In addition, study management provided two mandatory vicarious trauma trainings for the full protocol team. Of seven sites not already offering no-cost professional counseling to staff responding to GBV, five began doing so as a result of SOP implementation. Sites also shared experiences with providing GBV support, including lessons learned from referral provision and follow-up, and preventing vicarious trauma, via presentations during regular multisite HOPE team meetings. Debriefing and problem solving regarding individual and systemic challenges were also provided directly to counselors and site leadership by study management during site assessment visits.

### Process Evaluation Surveys

Anonymous process evaluation surveys were completed by 17 site leaders (1–3 respondents at each of the 14 sites) and 32 staff (1–5 staff at 13 of 14 sites) after SOP implementation.

**Fig. 1:**
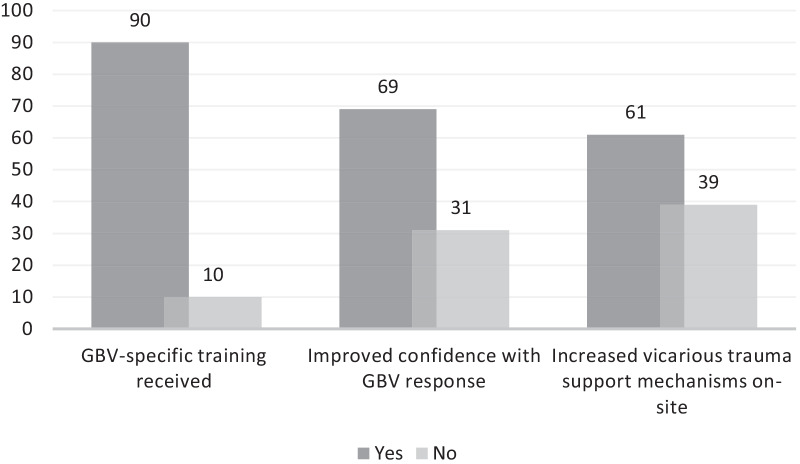
Staff-reported improvements after SOP implementation (% of respondents)

As illustrated in Fig. 1, most staff noted improvements in skills and resources available to them after SOP implementation, such as increased GBV-specific trainings, improved confidence with GBV response, and an increase of vicarious trauma support mechanisms available on-site, including professional counselors and psychologists from outside organizations.

“I am now spontaneous in my responses as I am sure of the information I am providing,” mentioned one counselor, continuing that “due to debriefing, I am able to give off my best at every intimate partner violence counseling session.” Improved support for staff was frequently mentioned, with counselors reporting that “I feel more supported by leadership and do not have grey areas,” and “debriefing with other counselors always helps with lifting the burden of being the carrier.” Site leadership also mentioned the importance of a supportive atmosphere, stating “the environment has to be welcoming from the onset… participants need to be clearly aware of the assistance they might get from the study team.”

**Fig. 2:**
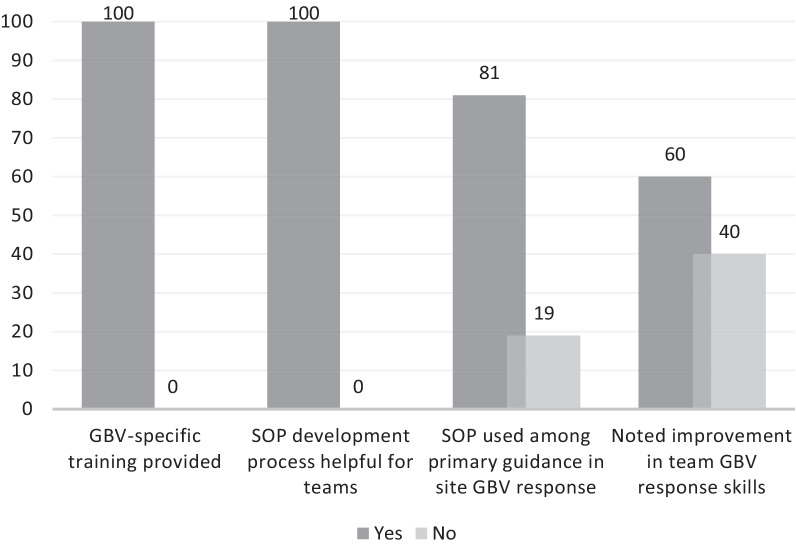
Site leadership-reported improvements after SOP implementation (% of respondents)

Post SOP implementation, site leadership respondents also reported improvements as illustrated in Fig. 2. Improvements were noted in team GBV response skills, utilization of the GBV Response SOP, and an appreciation of the SOP implementation process. Noted benefits to participants included the coordinated response: “IPV was mainly being addressed by clinicians and counselors—now the multi-disciplinary site team has a chance to participate in the management of IPV,” as reported by one IoR. One study coordinator noted that the SOP “makes it easier for the site staff to identify the victims and assist in helping them on time,” and a site leader reporting that this “has guided our management of IPV cases and ensured that we reviewed our referral network to ensure adequate referral was in place.” This site leader also appreciated the opportunity to conduct a “critical examination of the site preparedness to manage cases,” and the attention paid to preventing and addressing vicarious trauma. Finally, site leadership also mentioned increased knowledge and vicarious trauma prevention resources as benefits to their teams.

**Fig. 3:**
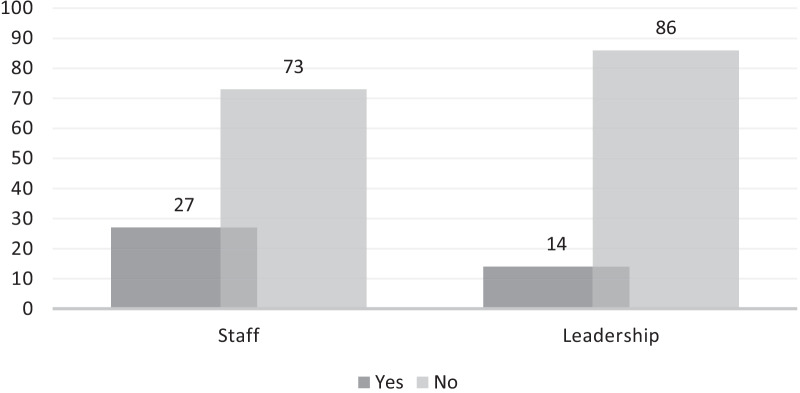
Improvements in referral networks after SOP implementation, as reported by staff and leadership (% of respondents)

Improvements related to referral networks as reported by both site staff and leadership were minimal, however (Fig. 3). Improvements noted by site leadership included improved relationships with referral organizations, smoother referrals and inclusion of additional referral organizations, with site staff mentioning the addition of transport to referral organizations, leadership follow-up on referral outcomes, and provision of referral service pamphlets. Both leadership and staff expressed that a lack of adequate referral organizations—such as those providing financial, legal, or housing support—remained a challenge when assisting participants. Site leadership also highlighted the importance of changing societal norms about GBV to improve its identification and response, with one sub-investigator noting, “women will only become more open about reporting when all degrees of IPV become socially/culturally unacceptable.”

Site leadership made multiple recommendations for SOP implementation in future trials across all study populations, including integration of GBV-related topics into all participant engagement activities and monitoring and documentation of all instances of GBV and resulting follow-up.

Although the SOP encouraged sites to invite organizations to PEAs, only two sites reported that a GBV service organization attended an event—both sites were in Zimbabwe and engaged the same organization. Overall, four sites, two each in South Africa and Zimbabwe, reported carrying out discussions related to GBV with participants during these events—two as a result of SOP implementation, with three of these sites addressing the topic of GBV multiple times. One counselor emphasized the importance of this collaboration: “the participants have managed to acquire more knowledge on how to take care of themselves as we now interact more with the support system since we invite them to address participants [in] retention meetings.”

## Discussion

Our experience in HOPE illustrates that the use of tailored SOPs to identify and respond to GBV is a feasible strategy to build standardized GBV services within research sites and potentially within other clinics providing HIV prevention services in a syndemic setting. SOP development can be informed by the use of survey tools and accepted best practices to create a tailored, systematic approach through which services for GBV survivors can be improved, and support systems and clinic atmospheres around GBV can be strengthened to benefit both staff and the populations they serve [39].

HOPE teams were able to utilize their SOPs and reported increased training, improved confidence, and more on-site support to prevent and address vicarious trauma. Leadership at research sites also reported improved response skills among their teams. Enhanced atmospheres of support for participants and staff were noted by site team members when asked to reflect on the SOP process.

The combination of needs and process evaluation surveys also allowed the HOPE team to identify ongoing gaps, such as limited time for providing counseling and follow-up to participants. Leadership reports of insufficient referral networks in the process evaluation, and limited engagement of referral organizations at PEA events, highlight the importance of adequate community-based referral networks and ongoing efforts to address societal norms that lead to GBV and represent challenges to the implementation of a GBV Response SOP. While clinic teams can be equipped to provide first-line support and offer a comprehensive network of warm referrals, survivor access to crucial services such as emergency housing and financial assistance, legal and protection services, and ongoing counseling is limited by what is available nearby [40]. Efforts to improve local referral networks have had success when referral organizations received advance training and iterative processes for maintaining referral relationships were implemented, however this was outside the scope of the HOPE study [41]. Furthermore, site services do not improve primary prevention of GBV, and these services may be hampered when GBV is normalized within a community, regardless of efforts to sensitize participants [42]. Our experience indicates that future efforts to improve GBV response would benefit from funding dedicated to professional counseling for site staff and building collaborations with primary prevention organizations.

Our assessment of the SOP process aimed to determine what impacts this process had on site GBV response. However, recall bias and social desirability bias due to the timing of the process evaluation and respondent knowledge that study management would have access to survey results may have impacted findings. Because leadership from one site did not respond to the initial needs assessment, it is possible that not all site needs were accounted for during the process. In addition, because the SOP development and implementation process was carried out to improve services for all participants rather than as part of the trial, participants were not randomized to SOP implementation or standard of care. Despite the importance of understanding participant experience, data were not collected regarding the SOP and its use or impact on participant wellbeing to avoid creating an undue data collection burden on participants while allowing for collection of data related to the primary study objectives. Therefore, indicators of improved counseling abilities and increasingly supportive site atmospheres are based on site team report. Collecting data on participant experiences, needs, and recommendations before and after the process, and devoting resources to understanding and applying learning from this data, would enhance SOP development and implementation in future similar efforts. Finally, there were few identified instances of GBV during HOPE, limiting our ability to explore how GBV response guided by SOP implementation related to HIV prevention and adherence to study product.

Our experience also suggests that supportive research staff and clinic environments for GBV survivors can be fostered as a complement to biomedical HIV prevention research services, even if extensive resources are not available. Specifically, systematically assessing the needs of research and clinic staff and working to address identified needs through standardized procedures is a low-cost process that can be applied in a variety of settings. Although more research is needed, studies suggest that delivering survivor-centered support to users of biomedical HIV prevention products may support them to continue using PrEP as long as they feel it is necessary, rather than discontinuing use or struggling with consistent use due to partner objection or trauma related to experiences of violence [6, 7, 43].

## Conclusions

The HOPE GBV Response SOP Template (Supplemental File 1) implementation experience contributes to ongoing efforts to expand GBV identification and support in research and service provision related to PrEP [35]. The process and experiences described here informed the development of the United States Agency for International Development-supported CHARISMA-CHOICE SOP and Job Aid for Addressing Partner Relationships and IPV in PrEP Services (Supplemental File 2), now publicly available [44]. More research is needed to understand the client experience with clinic teams utilizing a GBV response SOP and to further understand what works to support staff providing these services in resource-limited settings. Further investigation into best practices of GBV response teams may reveal additional tools that translate well to HIV prevention settings. We recommend that the approach described here be considered by PrEP implementers and researchers as an efficient and acceptable way to improve GBV identification and response.

## Supplementary Information

Below is the link to the electronic supplementary material.

Supplementary file1 (PDF 188 KB)—MTN-025/HOPE GBV Response SOP Template. Word document distributed to sites for adaptation and implementation during the MTN-025/HOPE study.

Supplementary file2 (PDF 436 KB)—CHARISMA-CHOICE SOP for Addressing Partner Relationships and IPV in PrEP Services. Word document version of the SOP developed by the CHOICE Collaboration, finalized in October 2020.

## Data Availability

Not applicable.

## Abbreviations

ASPIRE: A Study to Prevent Infection with a Ring for Extended Use
CHARISMA: Community Health Clinic Model for Agency in Relationships and Safer Microbicide Adherence
GBV: Gender-based violence
HIV: Human immunodeficiency virus
HOPE: HIV Open-label Prevention Extension
IOR: Investigators of record
IPV: Intimate partner violence
PEA: Participant engagement activity
PrEP: Pre-exposure prophylaxis
SOP: Standard operating procedure
WHO: World Health Organization
