# Supplementary material for: Integrating Gender-Based Violence Screening and Support into the Research Clinic Setting: Experiences from an HIV Prevention Open-Label Extension Trial in Sub-Saharan Africa
Source: AIDS Behav. Author manuscript; Available in PMC 2023 Apr 24. (PMC10036410; doi:10.1007/s10461-022-03864-6)
Supplement: HOPE GBV Response SOP Template [file NIHMS1883207-supplement-Template_SOP_for_IPV.pdf]

SOP No.: MTN-XXX, version 1.0
Title: Intimate Partner Violence & Sexual Assault Support and Follow-Up
Original Effective Date: XX MMM YYYY **Revision Effective Date:** Not Applicable

Purpose

To define procedures for identifying study participants who experience intimate partner violence or sexual assault (IPV/SA), and for providing these participants with adequate support, referral, and follow-up.

Scope

This procedure applies to all staff involved in support, referral or follow-up of participants who report IPV/SA for MTN.

Responsibilities

[MTN staff members who work directly with participants, including the clinic, administrative, and community teams] are responsible for understanding and following this SOP.

[MTN Study Coordinator or other designee] is responsible for training site staff to work with participants in accordance with this SOP and for day-to-day oversight and support of relevant staff.

[MTN Study Coordinator or other designee] is responsible for monitoring and assessing the effectiveness and efficiency of referral and support activities, and for working with study teams to improve strategies as needed to provide the best possible support participants.

MTN Investigator of Record has ultimate responsibility for ensuring that all applicable staff members follow this SOP.

Background & Definitions

MTN study teams are committed to providing effective support and referral for participants who experience intimate partner violence or sexual assault.

According to the World Health Organization, ***Intimate Partner Violence***, or IPV, refers to any behavior within an intimate relationship that causes physical, psychological or sexual harm to those in that relationship. It includes acts of physical aggression (slapping, hitting, kicking, or beating, for example), psychological abuse (intimidation, humiliation, and threats, for example), forced sexual intercourse or any other controlling behavior (including isolating a person from family or friends, monitoring their movements or restricting access to information or assistance, for example). Intimate partner violence also includes violence committed by former partners and individuals in dating relationships.

Sexual Assault, or SA, is any type of sexual contact or behavior that occurs without the explicit consent of the recipient. Sexual assault can be perpetrated by someone who is a stranger to the victim, and it can also occur within intimate partnerships, or friendships or familial relationships. Sexual assault includes a range of behavior from unwanted touching to forced intercourse. Examples of “force” used to commit sexual assault include but are not limited to, threats or intimidation, physical violence, or using drugs or alcohol to incapacitate a victim.

Procedures

1.0 Information Provision & Dissemination

1.1. Site teams should develop and maintain an accurate and current referral network of organizations that provide services to women and men who have experienced IPV/SA. Relevant services include but are not limited to: physical and mental health services; provision of post-exposure prophylaxis (PEP) and emergency contraception; shelter and relocation services; financial and group support; and protection, legal, and advocacy services.

1.1.1. Potential referral organizations should be contacted in advance of any first referral. Meetings between relevant site & organization staff should occur in order to determine, at minimum, the following: type and range of services provided; requirements and qualifications needed to receive services; preferred referral pathways; contact information; and any informational/publicity materials provided by the organization. Refer to [*Attachment X and/or reference relevant SOPs*] for a listing of referral providers.

1.1.2. Meetings with referral organizations and site staff should occur every *[site teams to determine specified time frames based on relevance of service, frequency of referrals, and likelihood of updates]* in order to update important information as needed. Site staff should also visit the organization as needed to collect publicity materials such as informational brochures or cards.

1.1.3. *[Site to add how further information gathered about referral organizations from initial and follow-up meetings, as well as participant feedback, will be captured and updated on site-specific referral documents]*

1.2. Site teams will develop methods to ensure that basic information on available services is available to participants upon request and as general information.

1.2.1. Informational materials from relevant organizations should be present in clinic rooms and site waiting areas *[sites to add applicable locations that are accessible and/or potentially more private for participants, such as restrooms or the pharmacy]*

1.2.2. Site staff should be generally familiar with referral organizations and aware of referral processes. *[Site to specify responsible persons]* will ensure that team members get relevant updates on this information by *[site to insert process and timeline for information sharing within the team]*

1.2.3. Team members most likely to hear participant disclosures of violence first-hand, such as *[site to insert likely staff]*, should be equipped to provide participants with information and referrals.

1.2.4. *[Site to insert possible educational activities for participants – for example, inviting referral organizations to give presentations in the waiting room or during participant engagement events]*

2.0 Identification, Provision of Support and Safety Planning

2.1. It is expected that some study participants may self-disclose experiences or fears of violence. For those participants who self-disclose, or if participants report IPV/SA within their responses to behavioral or other questionnaires, *[site to insert relevant staff]* should flag this for follow-up during the clinic visit. However, additional signs and symptoms should serve as possible indicators of IPV/SA, and clinicians and counselors should recognize and be particularly aware of the following symptoms:

- Mental health problems such as depression, generalized anxiety disorders, chronic fatigue and sleep disturbances
- Physical ailments such as chronic backache or headache
- Frequent injuries, bruising, or sprained/broken bones

2.1.2. Participants who self-disclose experiences of violence during conversation, questionnaire completion, or after further information-gathering during exams should be referred to *[site to insert relevant staff]* for first-line support, referral, and follow-up as indicated.

2.2. First-line support, which consists of active listening, validation of the participant's experience, and reflection and reframing to alleviate potential feelings of guilt or shame, will be provided to all participants who disclose IPV/SA.

2.2.1. While all site staff who have regular interactions with participants will be trained to respond in a survivor-centered way to disclosures of violence, *[site to insert specific team members, suggestions include counselors, nurses, behavioral interviewers, medical officers, and community team members]* will receive additional training to provide first-line support.

2.2.2. *[Site teams to detail how referrals within-site will occur when staff not trained in first-line support are approached by participants with concerns of violence]*

2.3. *[Site to insert relevant staff]* will provide clinical care to all participants who have recently experienced violence or an assault, or who have ongoing health concerns related to an experience of violence, according to WHO guidelines for Responding to intimate partner violence and sexual violence against women and within site capacity. Referrals will be conducted for all symptoms or complaints outside of site capacity to treat, according to processes outlined below in section 3.

2.3.1. Clinical care and referrals will take into account as much information about the event as the participant is comfortable sharing and include the offer of provision (or referral for) HIV post-exposure and STI prophylaxis and/or emergency contraception, according to local standard of care.

2.4. *[Site to insert relevant staff]* will provide immediate counseling and offer ongoing counseling or referrals for further counseling as needed. Counseling on-site will include discussion of healthy coping mechanisms, self-care, and safety planning where applicable, according to local standards and the WHO mhGAP Intervention Guide.

2.5. Participants who are thought to be at risk of further violence will also be guided through a safety planning process by *[site to insert key trained staff]*. The safety planning process can be informal, such as a verbal discussion of options available to the participant should she feel threatened, or more formal, including a written plan or action steps toward protection orders and/or temporarily or permanently leaving an abusive partner. Team members conducting safety planning will do so with the understanding that only the survivor is able to determine what is safe for her. At minimum, safety planning should be participant-driven and include:

- Discussion of possible escalation of violence and potential protective factors in the participant's life, such as supportive neighbors or family members
- Overview of resources and options available to the participant, including legal action such as protective orders
- Plan for a check-in with either a referral organization or the site, after an agreed upon amount of time
- Other items as relevant – refer to resources and guidance referenced in this SOP

2.6. *[Site to outline any relevant workshopping of possible scenarios – for example, mock participant visits or review of case studies]*

3.0 Referral & Follow-Up

3.1. Site staff to refer to *[site to insert appropriate SOPs, e.g. referral and/or safety SOPs]* for referral and documentation procedures. Special effort will be made to ensure the following:

- Referrals are participant-driven and agreed upon rather than based on staff perception of need
- Participants are accompanied as much as possible for referrals, such as accompanying participants for walk-ins or making referral calls in the presence of the participant *[site to insert relevant transport considerations as needed]*
- Confidentiality is prioritized over divulging unnecessary details of a participant's experience without her agreement

3.2. Site staff to note participant preferences for follow-up and contact participants accordingly. Those participants who agree to be contacted about the incident or experience should be contacted by the agreed-upon staff member(s), and ongoing agreement should be obtained for further follow-up. Participants who do not uptake referrals or do not agree to be contacted about the issue again will be reassured that resources are available for them in the future should they change their minds.

3.3. Working with violence survivors has the potential to increase the risk of experiencing vicarious, or secondary, trauma, particularly for service providers or staff who have themselves experienced violence. The site will endeavor to reduce the impact of vicarious trauma on staff through the following methods:

- The team will hold group de-brief sessions to discuss participant experiences, or general staff and site capacity to respond, monitored by *[site to insert relevant staff]* to identify learnings and potential improvements in site response. Sessions will maintain a participant-centered approach. *[site to insert frequency of sessions or outline how timing of sessions will be organized]*
- Site leadership *[or site to insert relevant staff]* will work with impacted staff on their needs, including referrals to relevant organizations for further support. *[site to outline any additional support to be offered to staff, such as one-on-one time with colleagues or individual debriefing sessions, as appropriate]*

List of Abbreviations and Acronyms

IPV	Intimate Partner Violence
SA	Sexual Assault
MTN	Microbicide Trial Network
SOP	Standard Operating Procedure
SSP	Study-Specific Procedures
PEP	Post-exposure prophylaxis
WHO	World Health Organization

[Insert additional as applicable]

Attachments

Attachment *X*: Responding to intimate partner violence and sexual violence against women, *WHO clinical and policy guidelines.*
Attachment *X*: WHO mhGAP Intervention Guide, 2010
Attachment *X*: IPV & SA SOP Implementation Plan
[List any additional as needed]

References

MTN-025 SSP Manual Section 11
MTN SOP for Care, Counseling, Support, and Referrals
MTN SOP for Safety Monitoring and AE Reporting
[List any additional as needed]

History

Version	Effective Date	Supersedes	Review Date	Change
1.0	*Xx Mon YR*	NA	*Xx Mon YR*	Initial Release

Approval

______________________________ _______________
Author, Author's Title Date

______________________________ _______________
Reviewer, Reviewer's Title Date

Attachment X: IPV & SA SOP Implementation Plan

[The below chart pinpoints site action items for any training or resources needed in order to begin implementing this SOP. This chart should be updated as action items are completed]

SOP Implementation Plan:

Action Item	Resources Available	Resources Needed	Person Responsible	Target Date for Completion	Date Completed & Comments
Reach out to potential referral partners to develop network	*ACFODE, FIDA, CEDOVIP*	*Protocol Team Training (Referral networks)*	*Study Coordinator, Community Team Leader*	*16 March 2017*	*16 March 2017 (complete but ongoing)*
