# Supplementary material for: Integrating Gender-Based Violence Screening and Support into the Research Clinic Setting: Experiences from an HIV Prevention Open-Label Extension Trial in Sub-Saharan Africa
Source: AIDS Behav. Author manuscript; Available in PMC 2023 Apr 24. (PMC10036410; doi:10.1007/s10461-022-03864-6)
Supplement: CHARISMA-CHOICE SOP and Job Aid for Addressing Partner Relationships and IPV in PrEP Services [file NIHMS1883207-supplement-SOP_for_Addressing_Partner_Relationships_and_IPV_in_PrEP_Services.pdf]

Standard Operating Procedure for Addressing Partner Relationships and Intimate Partner Violence in Pre-Exposure Prophylaxis (PrEP) Services

October 2020

Acknowledgments

This standard operating procedure (SOP) was developed by Michele Lanham, Robyn Dayton, and Rose Wilcher of FHI 360 and Elizabeth Montgomery and Miriam Hartmann of RTI International. Technical review of the SOP was provided by Amelia Peltz of the U.S. Agency for International Development (USAID), Thesla Palanee-Phillips of Wits RHI, Myra Betron of Jhpiego, and Heidi Cham and Meagan Cain of the U.S. Centers for Disease Control and Prevention.

This work was made possible by the generous support of the American people through USAID and the U.S. President's Emergency Plan for AIDS Relief (PEPFAR). It is the result of a collaboration between the Community Health Clinic Model for Agency in Relationships and Safer Microbicide Adherence (CHARISMA) project; the Meeting Targets and Maintaining Epidemic Control (EpiC) project; the Reaching Impact, Saturation, and Epidemic Control (RISE) project; and the Collaboration for HIV Prevention Options to Control the Epidemic (CHOICE) activity. The contents are the responsibility of CHARISMA, EpiC, RISE, and CHOICE and do not necessarily reflect the views of USAID, PEPFAR, or the United States Government.

CHARISMA is a five-year cooperative agreement (AID-OAA-A-14-00012) funded by PEPFAR through USAID. CHARISMA aims to promote women's agency to consistently and safely use antiretroviral-based HIV prevention methods and mitigate intimate partner violence. CHARISMA is led by RTI International with core partners FHI 360, Wits RHI, and the University of Washington.

EpiC is a global cooperative agreement (7200AA19CA00002) funded by PEPFAR and USAID. EpiC provides strategic technical assistance and direct service delivery to achieve HIV epidemic control and promote self-reliant management of national HIV programs by improving HIV case finding, prevention, treatment programming and viral load suppression. EpiC is led by FHI 360 with core partners Right to Care, Palladium International, Population Services International, and Gobee Group.

RISE is a global cooperative agreement (7200AA19CA00003) funded by PEPFAR through USAID. RISE works with countries to achieve a shared vision of attaining and maintaining epidemic control, with stronger local partners capable of managing and achieving results through sustainable, self-reliant, and resilient health systems by 2024. RISE is led by Jhpiego in collaboration with ICAP at Columbia University, Management Sciences for Health, ANOVA Health Institute, BAO Systems, Johns Hopkins University Center for Public Health and Human Rights, and Mann Global Health.

CHOICE is an 18-month collaboration funded by USAID, in partnership with PEPFAR, through EpiC and RISE. The goal of this partnership is to address technical gaps and support national scale-up of PrEP in PEPFAR countries through catalytic evidence generation, translation, and research utilization. CHOICE is led by FHI 360 and Jhpiego.

Suggested Citation:
FHI 360 and RTI International. Standard Operating Procedure for Addressing Partner Relationships and Intimate Partner Violence in Pre-Exposure Prophylaxis (PrEP) Services. Durham (NC); 2020.

Abbreviations and Acronyms

GBV	Gender-based violence
HIV	Human immunodeficiency virus
IPV	Intimate partner violence
PEP	Post-exposure prophylaxis
PrEP	Pre-exposure prophylaxis
SA	Sexual assault
SOP	Standard operating procedure
STI	Sexually transmitted infection
WHO	World Health Organization

Definitions

Sex — refers to the classification of people as male, female, or intersex or another sex based on a combination of sexual and reproductive organs, chromosomes, and hormones.

Gender — social ideas about what traits, roles, responsibilities, and behaviors are acceptable for people born with female or male biological characteristics. The social definitions of what it means to be male or female vary among cultures and change over time.

Gender identity — refers to a person's sense of self as being male, female, nonbinary, or another gender, which may or may not correspond to the sex assigned to them at birth.

Gender-based violence (GBV) — any harmful act that is perpetrated against a person's will and that is based on socially ascribed (i.e., gender) differences between males and females; used to maintain and reinforce power differences based on gender.

Intimate partner violence (IPV) — a form of gender-based violence that refers to any behavior within an intimate relationship that causes physical, psychological, or sexual harm to those in that relationship.[1] It includes:

- Physical aggression (slapping, hitting, kicking, or beating)
- Emotional/psychological abuse (insults, belittling, constant humiliation, intimidation, threats of harm, threats to take away children)
- Sexual violence (unwanted sexual comments or advances; forcing someone to have sex or perform sexual acts when they do not want to; nonconsensual sexual touching; nonphysical sexual acts such as sexting; harming someone during sex; forcing or pressuring someone to have sex without protection from pregnancy or infection)
- Economic abuse (use of money or resources to control an individual; blackmailing; refusing right to work; taking earnings; withholding resources as punishment)
- Other controlling behavior (including isolating a person from family or friends; monitoring their movements; or restricting access to financial resources, information, education, medical care, or other resources)

[1] World Health Organization (WHO). Violence info: intimate partner violence [Internet]. Geneva: WHO; 2017. Available at: https://apps.who.int/violence-info/intimate-partner-violence/.

IPV also includes violence committed by former partners and individuals in dating relationships. IPV also encompasses harmful practices such as female genital mutilation/cutting; child, early, and formed marriage; and dowry-related murders. IPV occurs in all settings and among all socioeconomic, religious, and cultural groups. The vast majority of victims of IPV are cisgender women and girls; however, IPV also occurs against transgender women and cisgender and transgender men. Lesbian, gay, bisexual, and transgender people are at particularly high risk of IPV.

Routine enquiry — an approach to identifying cases of IPV among all clients who present for specific services, without resorting to the public health criteria of a complete screening program.[2] It is recommended in certain services for populations that may be at a higher risk of experiencing violence, including antenatal care, HIV care and treatment, HIV testing services, and pre-exposure prophylaxis (PrEP) services. Routine enquiry is used in settings where clinical enquiry cannot be conducted but where violence is a known risk factor for HIV.[3]

Clinical enquiry — an approach to identifying IPV by staying attentive to possible clinical cues and other signs and asking about violence if you note these cues. Clinical cues include on-going stress, anxiety, or depression; substance misuse; thoughts, plans, or acts of self-harm or (attempted) suicide; injuries that are repeated or not well explained; repeated sexually transmitted infections (STIs) and unwanted pregnancies.[2]

First-line support — the minimum level of (primarily psychological) support and validation of their experience that should be received by all clients who disclose violence to a health care (or other) provider. First-line support involves five tasks, summarized by the acronym “LIVES” (listen, inquire about needs and concerns, validate, enhance safety, and support).

Cisgender — describes people whose sense of gender identity corresponds with their sex assigned at birth.

Transgender — describes people whose gender identity is different from the sex assigned to them at birth.

---

[2] WHO. Responding to intimate partner violence and sexual violence against women: Clinical and policy buidelines. Geneva: WHO; 2013. Available at: link.

[3] Peltz A. Gender equality and gender-based violence priorities for USAID’s PEPFAR programs. Slide set; 2019; Washington DC.

Background

> **Important Note:**
> The SOP and job aid **must** be adapted by each PrEP program to reflect local laws, policies, resources and procedures. The bracketed sections of this SOP should be completed based on local circumstances and standards. The SOP should be formatted using the program's standard template, including dating and assigning a version number at the time of adaptation. The SOP should be reviewed and signed-off on by the relevant staff and updated as needed after implementation.

This SOP is intended to be adapted by programs providing pre-exposure prophylaxis (PrEP) so that support staff can identify clients who are experiencing intimate partner violence (IPV) and provide appropriate violence response services. It also provides procedures and counseling messages to help clients decide whether to talk to their partner(s) about their PrEP use and strategies for using PrEP with or without their partner's knowledge. The PrEP Job Aid for Discussing Partner Relationships was developed to support the implementation of this SOP. It can be found at https://www.prepwatch.org/.

Evidence has shown that gender-based violence (GBV) may act as a barrier to accessing HIV services, including PrEP services. IPV, in particular, is associated with lower PrEP uptake[4] and adherence[5] and increased PrEP interruption.[6] To improve effective use of PrEP, especially among adolescent girls, young women, and members of key populations (men who have sex with men, sex workers, people who use drugs, and transgender people), USAID requires that all PEPFAR-funded PrEP services conduct routine enquiry for IPV with all clients. After conducting routine enquiry for IPV, staff must then offer appropriate support and referrals to IPV response services, per World Health Organization (WHO) clinical guidelines.

WHO clinical guidelines[2] state that the following minimum requirements must be in place before providers can ask clients about violence:

- A protocol/SOP for asking about violence
- A standard set of questions to which providers can document responses[7]
- Providers who are trained on how to ask about IPV
- Providers who are trained to offer first-line support when violence is disclosed. First-line support "refers to the minimum level of (primarily psychological) support and validation of survivors' experience that should be received" by those who disclose violence to a health care or other provider. It shares many elements with "psychological first aid" in the context of emergency situations. The WHO uses the acronym LIVES[8] to help providers deliver first-line support:
    - Listening with empathy

---

[4] Lanham M, Hartmann M, Palanee-Phillips T, Mathebula F, Wilson E, Wagner D, et al. The CHARISMA intervention to promote PrEP use, improve relationship dynamics and reduce IPV: intervention and methodological adaptations for an RCT and considerations for future scale up. Sexual Violence Research Initiative Forum. Oct 2019.r an RCT and considerations for future scale up. Sexual Violence Research Initiative Forum. Oct 2019.

[5] Roberts ST, Nair G, Baeten JM, Palanee-Philips T, Schwartz K, Reddy K, et al. Impact of male partner involvement on women's adherence to the dapivirine vaginal ring during a phase III trial: Disclosure to male partners, partner engagement with the study, and partner support of ring use. J Acquir Immune Defic Syndr. 2016; 73(3): 313-322.

[6] Cabral A, M Baeten J, Ngure K, et al. Intimate partner violence and self-reported pre-exposure prophylaxis interruptions among HIV-negative partners in HIV serodiscordant couples in Kenya and Uganda. J Acquir Immune Defic Syndr. 2018;77(2):154-159.

[7] USAID, Office of HIV/AIDS, Gender and Sexual Diversity Branch provides additional guidance noting that standard questions are required.

[8] WHO. Health care for women subjected to intimate partner violence or sexual violence: A clinical handbook. Geneva: WHO; 2013. Available at: link.

  - Inquiring about the client's immediate needs and concerns
  - Validating the client's experience
  - Assessing and helping Enhance the person's safety
  - Linking the client to other Support
- A private setting with confidentiality ensured where providers ask about IPV
- A process for offering referrals or links to other services

When adapted to the local context, this SOP helps PrEP programs meet the requirement for having an SOP in place for conducting routine enquiry for IPV. The SOP also outlines how programs can ensure compliance with the other minimum requirements.

Purpose

This SOP defines procedures for using routine enquiry to identify PrEP clients who are experiencing IPV and for providing clients who disclose violence with adequate first-line support, referral, and follow-up. It provides staff with basic counseling tips for clients who are using PrEP or considering using PrEP in the context of an abusive relationship.

Scope

This procedure applies to all program staff involved in PrEP services (including health care workers, clinic support staff, and outreach workers who collaborate with the clinic) as well as staff who are involved in the support, referral, or follow-up of clients who report IPV (including social workers, support group leaders, and counselors). It is limited to creating an enabling environment to conduct routine enquiry, taking steps to identify individuals experiencing violence, and then providing "first-line support" to those who disclose violence. The final step of first-line support is appropriate referral, to both clinical and nonclinical services.

This SOP does not cover provision of comprehensive clinical services that should be available within the clinic or via referral (e.g., treatment of injuries, emergency contraception, post-exposure prophylaxis [PEP] for HIV and sexually transmitted infections (STIs), mental health screening and treatment for depression and post-traumatic stress disorder, and forensic examination). It also does not cover the provision of nonclinical services to which a client may be referred. For more information on clinical services, please see *Responding to Intimate Partner Violence and Sexual Violence against Women: WHO Clinical and Policy Guidelines*[2] and *Health Care for Women Subjected to Intimate Partner Violence or Sexual Violence: A Clinical Handbook*.[8] For more on nonclinical services, please see Section 8.2: Establish Coordination and Referrals between Health Services and Services of Other Sectors in *Strengthening Health Systems to Respond to Women Subjected to Intimate Partner Violence or Sexual Violence: A Manual for Health Managers*.[9]

Responsibilities

- All staff who interact with PrEP clients are responsible for understanding and following this SOP.
- The referral liaison is responsible for establishing and maintaining the referral network.
- [Program to fill in appropriate position(s)] is responsible for training program staff to work with clients in accordance with this SOP and for day-to-day oversight and support of relevant staff.

---

[9] WHO. Strengthening health systems to respond to women subjected to intimate partner violence or sexual violence: a manual for health managers. Geneva: WHO; 2017. Available at: link.

- Program staff who provide PrEP counseling should be trained to conduct IPV routine enquiry and provide first-line response.
- All program staff who directly interact with clients including [counselors, clinicians, clinic support staff, and community workers] should be trained to provide first-line response to clients who spontaneously disclose violence.[10]
- [Program to fill in appropriate position(s)] is responsible for monitoring and assessing the effectiveness and efficiency of routine enquiry; IPV identification; provision of first-line support, referral and support activities; and for working with program staff to improve strategies, including through supportive supervision, as needed to provide the best possible violence-related support to clients, as outlined in this SOP.

[Program to fill in appropriate position(s)] has **the** ultimate responsibility for ensuring that all applicable staff members follow this SOP.

Procedures

1.0 Preparation

1.1 Review local laws to determine the obligations of the health system to care for survivors, including female, male, and transgender survivors, and to understand any situations in which mandatory reporting of violence is required. A checklist of local laws is available in Job Aid 6.1 of *Strengthening Health Systems to Respond to Women Subjected to Intimate Partner Violence or Sexual Violence: A Manual for Health Managers*.[9]

1.2 Develop and maintain a referral network. An accurate and current referral network of local organizations that provide services to people who have experienced violence should be developed and maintained. Relevant services are listed in Appendix C: Referral Network Template. See Appendix B: Steps for Establishing and Maintaining a Referral Network.

1.2.1 Staff awareness of referral network. Program staff should be generally familiar with referral organizations and aware of referral processes. [Clinic to specify responsible people] will ensure that team members get relevant updates on this information by [insert process and timeline for information sharing within the team].

1.2.2 Supplemental information. Make informational materials from relevant organizations available in clinic rooms and waiting areas [Clinics to add applicable locations that are accessible and/or potentially more private for clients, such as restrooms or the pharmacy].

1.3 Create private spaces within the facility where no other clients or staff can hear the conversation.

1.4 Train staff who are conducting routine enquiry on first-line support (also known as LIVES) according to World Health Organization standards.[2]

1.4.1 It is recommended that all program staff, including peers and other community workers, who directly interact with clients should be trained on LIVES using the relevant portions of *Caring for Women Subjected to Violence: A WHO Curriculum*

---

[10] WHO. Caring for women subjected to violence: a WHO curriculum for training health-care providers. Geneva: WHO; 2019. Available at: link.

for Training Health-Care Provider[10] in order to ensure that all PEPFAR-funded programs are able to correctly, consistently, and compassionately respond to disclosures of violence. While this approach was designed to address the needs of cisgender women, the general steps for routine enquiry and the provision of first-line support are relevant beyond this population. Where there is a need for adaptation to address the unique experiences of men or transgender people, this is noted. For training materials that include examples tailored to key populations, see the health care worker training in the *LINKAGES Violence Prevention and Response* series of resources.[11]

1.5 Convene all the staff to review and discuss the SOP and ensure they know their roles, responsibilities, and have the appropriate coordination mechanisms in place to implement the SOP.

1.6 Tailor routine enquiry questions. Questions may need to be adapted based on the local language or terminology to describe forms of IPV. In addition, if you are working with key populations or translating questions into a local language, work with staff and beneficiaries to adapt the questions in Appendix A as needed to ensure the questions are clear and relevant to the experience of the target populations. This can be accomplished through conducting focus groups or more informal discussions with members of the target population(s).

1.7 Ensure all forms required by the program and by local policies, such as police referral and forensic forms as relevant, are available, and safe information storage procedures are in place. See Annex 11: Privacy and Confidentiality in Documentation in *Strengthening Health Systems to Respond to Women Subjected to Intimate Partner Violence or Sexual Violence: A Manual for Health Managers.*[9]

1.8 Develop or identify a system to support staff working with survivors. Depending on the program resources and staff preferences, this may include identifying external mental health service providers to whom program staff can disclose their concerns or identifying internal mental health providers who feel comfortable counseling their colleagues. See Section 5 for more.

1.9 Put systems in place that make refilling PrEP easy in cases where a partner throws away PrEP pills or forces a PrEP user out of the house without their belongings.

1.9.1 Sensitize program staff to the possibility of clients coming in or calling to request refills before the refill date.

1.9.2 Establish community refills.

---

[11] Dayton R, Morales GJ, Dixon KS. LINKAGES health care worker training: preventing and responding to violence against key populations. Durham (NC): FHI 360, 2019. https://www.fhi360.org/resource/linkages-violence-prevention-and-response-series.

2.0 IPV Routine Enquiry

2.1 Mandatory reporting. Based on the legal review conducted as part of preparation, program staff must communicate any limits to confidentiality, such as mandatory reporting requirements, to each client **before** inquiring about violence. The staff should tell the client what acts [program to include the relevant situations according to local laws such as violence with a firearm or harm to minors or the elderly] require the staff to report what is shared with them and to whom the report would be made.

2.1.1 If the client shares an experience that requires mandatory reporting, the provider should follow all local procedures for reporting and let the client know what will be done.

2.2 Routine IPV enquiry process.

2.2.1 [Insert program staff member(s) who will be responsible for routine enquiry] will conduct routine enquiry with all clients who are considering PrEP or currently taking PrEP. This can occur only if the client is alone or accompanied by a child under the age of two.

- It is important that clients engaged in PrEP services are asked about violence each time they visit the clinic for PrEP-related services. A client may have experienced violence in an existing relationship since her last visit, or she may have experienced violence from a new partner. In addition, the client may be seeing a different staff person than the last clinic visit.

2.2.2 When bringing up the issue of violence, the staff person should explain why questions about violence are being asked—concern for well-being of client as well as impacts of violence on HIV-related outcomes and PrEP use. They should also share that these questions are asked of everyone, and that many people experience problems at home. Explain the limits of confidentiality **before** asking the questions.

- To ensure that clients who have been asked about violence previously do not feel that their answers were disregarded, when violence is being addressed in later visits (after PrEP initiation), the provider should preface questions about violence by adding: “Partner relationships can affect PrEP use and your overall well-being. There may have been changes in your relationships since last time you came to the clinic, so we ask about any abuse in partner relationships each time you come to the clinic for PrEP, regardless of previous responses.”

2.2.3 Use a standard set of questions to ask about violence, such as those below, adapted from PEPFAR guidance:[12]

- Has your partner ever made you feel afraid, bullied or insulted you, threatened to hurt you, or tried to control you (for example, not letting you go out of the house)?

---

[12] These questions were adapted from the “Partner Information Form,” referenced in the PEPFAR 2020 Country Operational Plan Guidance for all PEPFAR Countries, which includes an illustrative set of IPV screening questions. Available at: link.

- Has your partner ever hit, kicked, slapped, or otherwise physically hurt you?
- Has your partner ever forced you into sex or forced you to have any sexual contact you did not want?

2.2.4 If working with key population members, consider developing questions tailored to their experiences (see Appendix A).

2.2.5 Do not pressure someone to disclose violence even if you believe it is occurring. Instead, remind those who do not disclose violence that you and the clinic are there to provide support in the future if violence occurs.

2.2.6 No service should ever be denied to a client because they did not disclose violence.

2.2.7 Regardless of whether a client discloses violence, they should still be offered PrEP unless post-exposure prophylaxis (PEP) would be more appropriate.

- If a client discloses sexual violence that includes a potential exposure to HIV within the last 72 hours, they should be offered PEP following the guidance found in Section 2.4 of *Health Care for Women Subjected to Intimate Partner Violence or Sexual Violence*.[8] After using PEP, if the client chooses to do so, they should be invited to come back to the facility to initiate PrEP. PEP use is considered a potential gateway for PrEP initiation.[13]

2.2.8 Additional counseling on the safe and effective use of PrEP should be provided. See Section 4. This is mandatory for clients who have disclosed violence but can be helpful for all clients.

3.0 Provision of First-line Support to Clients Who Disclose Violence

3.1 First-line support. If the client discloses violence during routine enquiry, program staff should provide first-line support, which includes basic counseling or psychosocial support. The WHO defines "first-line support" using the acronym "LIVES", which consists of:

**L**ISTEN	Listen to the client closely, with empathy, and without judging.
**I**NQUIRE ABOUT NEEDS AND CONCERNS	Assess and respond to the client's various needs and concerns—emotional, physical, social, and practical (e.g., child care)
**V**ALIDATE	Show the client that you understand and believe them. Assure the client that they are not to blame for the violence they have experienced.
**E**NHANCE SAFETY	Discuss a plan to protect from further harm if violence occurs again.
**S**UPPORT	Support the client by helping them connect to information, services, and social support.

[13] Baggaley, R. et al. (2015) The Strategic Use of Antiretrovirals to Prevent HIV Infection: A Converging Agenda. Clinical Infectious Diseases Supplement on HIV Postexposure Prophylaxis. Available at: link.

See *Health Care for Women Subjected to Intimate Partner Violence or Sexual Violence: A Clinical Handbook,*[8] Part 2: First-Line Support for Sexual Assault and Intimate Partner Violence for detailed information about implementing first-line support using LIVES. If the client will be using PrEP, during safety planning (step "E"), include PrEP as an item to pack if the client has to leave their home.

3.2 Additional information on referrals. In addition to the guidance on making referrals (as part of the 'Support' step in LIVES) on pages 29–31 of the WHO clinical handbook, the following is recommended. When making referrals, staff should only share the information that the client has agreed can be shared; all other information about the client must be kept confidential.

3.2.1 Accompaniment. When possible, [insert relevant staff] should accompany clients for walk-ins [insert relevant transport considerations as needed]. Provide the referral agency a referral letter (see Appendix D), including a detailed reason for referral (only if the client gives permission for this information to be included in the referral letter). This is an important option to offer the client because it can help the referral agency know the client's general situation and needs without the client having to re-count their experiences of violence.

3.2.2 Other active/warm referrals. In the absence of accompaniment, other active referral must be offered. This can include offering to help the client make an appointment by calling for them (ask the client in advance what information about their experience or needs should be shared), making a call with them, or offering a private place where the client can make a call. Offer the client a completed referral letter (see Appendix D) to take to the referral agency. In this situation, the reason for referral should be more general such as "the client is being referred for additional counseling" or "the client is being referred for health services" to reduce the risk of harm if someone else found the letter.

3.2.3 Offering printed copies of referral list. Program staff can offer printed copies of the referral list to clients, if safe and appropriate.

3.2.4 Note that sometimes having a referral letter or a referral list may put a client in danger if their partner finds it. Alternatively, you could offer to send it to the client by WhatsApp or email, if possible. Be sure to assess the client's comfort and safety to accept a referral letter or list.

3.2.5 Referral follow-up. Program staff should note client preferences for follow-up (phone or in person), in particular considering the means of follow-up that the client thinks is safest. Those clients who agree to be contacted should be contacted by the agreed-upon staff member(s) to determine whether the client received the services for which they were referred and what their experience was like at the referral organization. This will be documented [insert clinic's referral documentation procedures and forms]. Clients who do not uptake referrals or do not agree to be contacted about the issue again will be reassured that resources are available for them in the future should they change their minds.

3.3 Spontaneous disclosures of violence. It is expected that some clients may disclose experiences or fears of violence, without being asked about violence, to program staff including community workers.

3.3.1 All program staff involved in PrEP services (including health care workers, clinic support staff, and outreach workers who collaborate with the clinic) as well as staff who are involved in the support, referral, or follow-up of clients who report IPV (including social workers, support group leaders, and counselors) should be trained in first-line support.

3.3.2 If a staff person receives a spontaneous disclosure, they should be prepared to thank the client for sharing, provide information on the services available to survivors, and offer to go with the client back to the clinic (or provide information to allow for connection via phone) to link them with someone who has been trained on LIVES.

3.3.3 When a spontaneous disclosure is made and the conversation can occur privately, the person should listen empathetically, inquire about their immediate needs and concerns, and validate their experience (L-I-V in LIVES).

- If a staff person who has expertise in counseling clients on IPV (such as a therapist or social worker) is available, the program staff who initially receives the spontaneous disclosure may listen, inquire, and validate; then offer to accompany the client to the more experienced staff person who can complete E (enhancing safety) and S (link to support) in LIVES. This may be appropriate when the person receiving the spontaneous disclosure does not have time to go through all the steps of LIVES. However, the final decision as to whether the client will receive steps E and S from an additional staff person with more expertise must be made by the client. To limit the need for clients to repeat themselves, the person who receives the spontaneous disclosure should offer to summarize what the client disclosed for the staff person with more expertise.
- If the staff person with more expertise is not available or if the client does not want to talk to that staff person, the program staff receiving the spontaneous disclosure should cover all steps of LIVES.

4.0 PrEP Counseling[14]

4.1 Ask all clients, “Are you afraid that your partner would have a negative or violent reaction if they knew you were using PrEP?”

4.2 If a client discloses violence or indicates their partner could have a negative or violent reaction to the client using PrEP, discuss with the client about how to use PrEP safely in the context of their relationship.

4.2.1 If the client discloses violence or fears violence from their partner, ensure first-line support is provided (see Section 3.0).

4.3 Even if a client has not disclosed violence or fear of a partner, this counseling can help the client think about how to use PrEP in the context of their relationship. If feasible, it is recommended that this counseling be delivered to all clients in locations with a high prevalence of IPV or in situations, such as the COVID-19 pandemic, when IPV is increasing.

---

[14] These counseling messages are adapted from The CHARISMA Toolkit: An empowerment counseling intervention to improve women’s adherence to oral PrEP; RTI International, FHI 360, Wits RHI; 2020, forthcoming on PrEPWatch.org.

4.4 Counseling introduction:

[*For clients who have disclosed violence or fear violence from their partner*]

- People in abusive and controlling relationships are often more vulnerable to HIV. It may be difficult to negotiate condom use and to know your partner's status. This makes HIV prevention tools like PrEP even more important but taking PrEP can be a challenge in these circumstances. Clients experiencing abuse or control in their relationship may find it more difficult to take PrEP as prescribed and may need extra support. Let us brainstorm specific challenges you may face and together we can come up with ideas for overcoming these challenges.
- [*If discussion of whether to talk to one's partner about PrEP does not come up in the discussion of challenges, ask the following*]: PrEP can help people in abusive and controlling relationships protect themselves from HIV because they can use it without telling their partner. It may be challenging, but many people successfully use oral PrEP without telling their partners.
- However, if you would like to tell your partner, I can help you brainstorm ways to do so more safely, including having your partner speak to a staff person here.

[*For all other clients*]

- It's up to you to decide whether you want to talk with your partner about PrEP. Many PrEP users find it easier to use PrEP if their partner knows they are using it and is supportive. Other PrEP users want to use it without their partner knowing and are able to do that successfully. I would like to talk with you about whether you want to talk with your partner about PrEP and ways that you could use PrEP successfully. Is that ok?

4.5 Counseling topics:

[*For all clients*]

- Discuss their concerns about using PrEP safely.
  - Ask what they think their partner's response would be if they told their partner about PrEP.
  - Ask what they think their partner's response would be if their partner discovered they were using PrEP without them knowing.
- Discuss whether to tell their partner they are using PrEP
  - It is up to the client to decide whether to talk to their partner about PrEP. The client is the expert in their own relationship.
  - Ask how they feel about talking to their partner about oral PrEP. Do they want to talk to their partner about it?
  - Affirm their choice and then share "Tips for telling a partner" or "Strategies to take PrEP without their partner knowing."

[*If the client wants to tell their partner about PrEP*] Tips for telling a partner about PrEP use.

How to tell your partner

- Use clear and simple language

- Maintain eye contact, remain confident and calm
- Have prepared answers for anticipated questions
- Listen objectively to your partner's concerns
- Avoid blaming others for why you decided to use PrEP
- Observe your partner's body language

Where to tell a partner

- If you are afraid of a violent reaction, choose a location where you can exit safely if need be
- Don't be too far away from others so that you can get help if you need it
- Try not to have children present, and consider having a trusted person present

When to tell a partner

- When you will have enough time to say everything you need to say
- When you will have enough time for your partner to respond and ask questions
- When both of you are in a good mood and with a settled mind
- When neither of you is under the influence of drugs or alcohol

Additional tips from clients with experience telling their partners about PrEP:

- Talk about PrEP generally to see what they say before telling your partner you are using it
- Give a little information at a time
- Only tell your partner what they need to know (e.g., that you will be taking a pill each day to protect your health)
- If your partner is resistant at first, continue bringing it up over time until they become more supportive

[If the client does not want to tell their partner about PrEP] Discuss strategies to take PrEP without their partner knowing. These strategies may also be useful for clients whose partner found out they were using PrEP and were not supportive but the client wants to keep using it anyway.

- Store pills in places their partners will not look, such as a handbag, a keychain with storage, or with pads and tampons.
- Ask a neighbor or a nearby friend to keep the pills, although this can make it challenging to remember to take them as prescribed. If multi-month PrEP dispensing is available, the client could keep one month of PrEP in a safe place at their own home and the rest of the PrEP at a friend's house.
- Store a few doses in an unmarked container (ensure that this container is not clear plastic as sun can damage medication).
- If their partner monitors them closely, think of a reason for the regular clinic visits (e.g., explain that it is for another medical condition)
- Discuss explanations for any PrEP side effects they are experiencing or may experience.
- When their partner leaves the house, they can take a PrEP pill for that day out of the pill bottle or pill box and put it in a tissue in their pocket or handbag to take at the scheduled time (it should be taken at roughly the same time each day). Taking it from the tissue can be more discrete if the client's partner is home when it is time to take it. However, if they still think this may put them at too much risk, they can

take PrEP while their partner is out of the house even if that means taking it at a slightly different time each day.

- If the client thinks it will be difficult to use without their partner knowing or fears a violent reaction if the partners discovers they are using it without their partner knowing, the client could go ahead and tell the partner about starting a new medication for another medical condition (e.g., period pains, contraception, headaches, etc.)

[If the client doesn't want to tell their partner about PrEP] Brainstorm what to do if the partner finds the client's PrEP and becomes angry. The strategies above may also be useful for helping clients who want to keep using PrEP.

- Brainstorm with the client what to do if they need an emergency re-supply because their partner throws the PrEP away or the client has to leave home suddenly without their PrEP. Brainstorming this ahead of time could reduce barriers to getting an emergency re-supply if the client needs it. Ideally, community re-supply would be an option (see preparation).

[For all clients] End conversations on PrEP initiation or overcoming challenges to using PrEP by

- Helping the client identify their main reason for wanting to take PrEP and reminding the client of their strength and power.
- Asking the client who else in their life can support their PrEP use. Brainstorm ways this person could help the client.
- Asking the client what kind of support they need from the clinic to use PrEP safely and effectively.
- Letting the client know that you are available as a resource in the future if they struggle with IPV or with using PrEP as prescribed.
- If the client is not able to use PrEP as prescribed, discuss other options for reducing vulnerability to HIV.

5.0 Staff Experiences of Vicarious Trauma

5.1 Definition of vicarious trauma. Working with violence survivors can increase the risk of staff experiencing burnout and vicarious trauma, which is defined as the transformation of the health care provider's inner experiences as a result of empathetic and/or repeated engagement with violence survivors.[15] Staff may notice their beliefs about the world start to change after working with violence. For example, they may stop being able to believe that any relationship can be healthy. This is particularly a concern for staff who have themselves experienced violence. The clinic will work to reduce the effect of vicarious trauma on staff in the following ways:

---

[15] Adapted from Way I, VanDuesen KM, Martin G, Applegate B, Jandle D. Vicarious trauma: a comparison of clinicians who treat survivors of sexual abuse and sexual offenders. Journal of Interpersonal Violence. 2004 Jan; 19(1):49-71. Available at: link

5.1.1 Debrief sessions. The team should hold group de-brief sessions to anonymously discuss client experiences, or general staff and clinic capacity to respond, monitored by [insert relevant staff] to identify learnings and potential improvements in response. [Insert frequency of sessions or outline how timing of sessions will be organized.] If case-conferences are used to discuss challenging situations, these meetings can also include time to discuss the well-being of staff members involved in the case.

5.1.2 Supportive supervision. Those who supervise staff working with survivors should check in with staff about their well-being and their feelings about the work with survivors during regularly scheduled supervisory meetings. Supervisors should also ensure that staff know the services available to them and ask whether the staff members would like support.

5.1.3 Referral. Clinic leadership [or insert relevant staff] should work with staff members who have been affected about their needs, including referrals to relevant organizations for further support. [Insert any additional support to be offered to staff, such as one-on-one time with colleagues or individual debriefing sessions, as appropriate.]

5.1.4 Additional paid leave time. If possible, give additional paid leave time to staff who are experiencing vicarious trauma.

6.0 Documentation

Document the reporting of IPV and provision of care and referrals in the following places [clinic to adapt to their reporting procedures]:

- Referral log. Including initial referral and follow-up information.
- The GEND_GBV PEPFAR MER indicator should be used in clinics with GEND_GBV targets to document the number of clients who receive post-violence care as well as the type of violence that was reported (sexual or emotional/physical) and PEP provision and completion as relevant.

7.0 Adapted Procedures Due to COVID-19

7.1 IPV during COVID-19. During COVID-19, the risk of IPV has increased. At the same time, many violence response services have been forced to change their operations, which impacts the services available and how they can be accessed.

7.2 Key messages for clients about IPV during COVID. Talk to clients about the increased risk of violence occurring during COVID-19 and remind them they may visit or call the clinic during operating hours – or the clinic's after-hours phone number — to be linked to violence response services.

7.3 Client follow-up. For clients already identified as experiencing violence, arrange for safe follow-up via phone to help them make a plan to stay safe at home during lockdown or while living in quarantine. This should include tips on how to safely access support.

7.3.1 Safe communication. If program staff call clients who previously disclosed violence to follow up, establish a safe/code word that helps the survivor end a call quickly or alerts the program staff member to the need to change topics. Calls to the survivor should only be made if they have indicated this can be done safely and may include special instructions, such as "not identifying the organization that the program staff is associated with."

7.4 Update local referral directory more often than normal following the steps outlined in Appendix B: Steps for Establishing and Maintaining a Referral Network. Ask about changes in hours that services are available and for any advice on safe transport during lockdown/curfews so that this can be communicated to survivors.

7.5 PrEP counseling. Counseling for anyone who discloses violence should include finding ways to discreetly and safely take PrEP during quarantine or lockdown.

7.6 Resources for staff. Provide additional phone/internet credit to program staff tasked with responding to violence, such as psychologists or GBV focal points, as they make their services available virtually.

Appendices

Appendix A: IPV Routine Enquiry Questions for Key Populations
Appendix B: Steps for Establishing and Maintaining a Referral Network
Appendix C: Referral Network Template
Appendix D: Referral Letter Template

Appendix A: IPV Routine Enquiry Questions for Key Populations (to be adapted as needed for your local context)

These adapted IPV Routine Enquiry Questions include additional questions (1a-d) about forms of IPV that members of key populations may experience. If you further adapt these questions for your context, ensure that the questions:

- Provide specific examples of violent actions instead of simply asking, “Has a partner been violent?” as people understand “violent” to mean different things, and
- Are direct instead of general, such as, “How is your relationship?” which may be interpreted in different ways.

1. Has your partner ever made you feel afraid, bullied or insulted you, threatened to hurt you, or tried to control you (for example, not letting you go out of the house)?
   a. [For men who have sex with men, transgender women, sex workers]: Has your partner ever called you names, used slurs against you, or threatened to out you?
   b. [For men who have sex with men and transgender women]: Has your partner ever criticized your sexual performance, criticized your clothing, or asked you to act more masculine?
   c. [For transgender clients] Has your partner tried to control your transitioning process?
   d. [For transgender clients] Has your partner ever told you that no one else would want to be with someone like you because you are transgender?

2. Has your partner ever hit, kicked, slapped, or otherwise physically hurt you?

3. Has your partner ever forced you into sex or forced you to have any sexual contact you did not want?

Appendix B: Steps for Establishing and Maintaining a Referral Network

1 **Identify referral liaison.** Clinic leadership should identify a point person — the referral liaison— who is responsible for establishing and maintaining contact with referral organizations.

2 **Identify a diversity of services.** Efforts should be made to include organizations offering the services listed in Appendix C: Referral Network Template. Identify as many of these services as possible, but some services may not be available.

3 **Local standard of care for sexual assault.** Review the Ministry of Health guidelines. Review *Health Care for Women Subjected to Intimate Partner Violence or Sexual Violence: A Clinical Handbook*[7] Part 3: Additional Care for Physical Health after Sexual Assault and identify the local standard of care for recent victims of sexual assault, including HIV PEP, STI prophylaxis, emergency contraception, and forensic testing. Determine which of these services are available at your clinic and to which services clients will need to be referred.

4 **Preliminary contact.** Potential referral organizations should be contacted in advance of any first referral. Meetings between the referral liaison and a point person at the referral organization should occur in order to determine, at minimum, the following: type and range of services provided, requirements and qualifications needed to receive services, preferred referral pathways, contact information, and any informational/publicity materials provided by the organization. Meetings should also assess whether the referral organization provides survivor-centered, stigma-free services and what services are available for specific key population members, citizens, noncitizens, and asylum seekers. Referral organization names, addresses, contact details, and populations served will be listed in a completed version of Appendix C: Referral Network Template.

5 **Assess and provide training requirements**. If an organization that has historically served women in the general population is open to serving key population members but has not been sensitized on how to do so, training on the unique needs of key populations should be provided to the organization's focal point.

6 **Establish linkage relationship.** The referral liaison should meet with the point person at each referral organization every [program staff to determine specified time frames based on relevance of service, frequency of referrals, and likelihood of updates] to maintain a working relationship with the referral organizations and update important information as needed. Program staff should also visit the organization as needed to collect publicity materials such as informational brochures or cards.

7 **Update referral network.** Referral networks should be updated at least annually.

8 **Establish technical working group (if feasible).** One way to continually gather information about referral organizations is to convene all local organizations that offer violence response services on a regular basis (either virtually or in person) in a technical working group dedicated to a coordinated violence response. These organizations can provide updated information on the services they currently offer and any changes to the services they offer. The technical working group can also be attended by community representatives and/or individuals who accompany victims to services. These representatives can share anonymous feedback from those who have sought services and highlight issues such as poor treatment of survivors. Those who offer those services can describe the activities that will be undertaken to address any issues, or a decision can be made to remove this service from the list of referrals available. After each meeting of this group, the clinic can update the referral directory according to the changes described. This information should be shared with all program staff as part of regular staff meetings.

Appendix C: Referral Network Template

COMMUNITY SERVICE ORGANIZATIONS – **HEALTH SERVICES**

Includes post-exposure prophylaxis (PEP), forensic exams, family planning, emergency contraception, STI screening and treatment, OB/GYN, mental health screening and treatment, psychological support/counseling, substance abuse treatment

**[Organization/Facility Name]**<br>Phone number:<br>Fax:<br>Email:<br>Address:<br>Hours:<br>Services offered:<br>Populations served:<br>Send referral letters by: [phone/email/hard copy/fax]	**[Organization/Facility Name]**<br>Phone number:<br>Fax:<br>Email:<br>Address:<br>Hours:<br>Services offered:<br>Populations served:<br>Send referral letters by: [phone/email/hard copy/fax]	**[Organization/Facility Name]**<br>Phone number:<br>Fax:<br>Email:<br>Address:<br>Hours:<br>Services offered:<br>Populations served:<br>Send referral letters by: [phone/email/hard copy/fax]

COMMUNITY SERVICE ORGANIZATIONS – **SOCIAL SERVICES**

Includes gender-based violence services, child protective services, psychosocial support including crisis counseling and support groups, women's groups, organizations working with marginalized or special needs populations, child care, housing/shelters, transportation assistance, food assistance, employment training and financial aid

**[Organization/Facility Name]**<br>Phone number:<br>Fax:<br>Email:<br>Address:<br>Hours:<br>Services offered:<br>Populations served:<br>Send referral letters by: [phone/email/hard copy/fax]	**[Organization/Facility Name]**<br>Phone number:<br>Fax:<br>Email:<br>Address:<br>Hours:<br>Services offered:<br>Populations served:<br>Send referral letters by: [phone/email/hard copy/fax]	**[Organization/Facility Name]**<br>Phone number:<br>Fax:<br>Email:<br>Address:<br>Hours:<br>Services offered:<br>Populations served:<br>Send referral letters by: [phone/email/hard copy/fax]

COMMUNITY SERVICE ORGANIZATIONS – **LEGAL SERVICES**

Includes legal aid (representation and provision of information), police/law enforcement, child protective services, local courts, prosecutor's office

**[Organization/Facility Name]**<br>Phone number:<br>Fax:<br>Email:<br>Address:<br>Hours:<br>Services offered:<br>Populations served:<br>Send referral letters by: [phone/email/hard copy/fax]	**[Organization/Facility Name]**<br>Phone number:<br>Fax:<br>Email:<br>Address:<br>Hours:<br>Services offered:<br>Populations served:<br>Send referral letters by: [phone/email/hard copy/fax]	**[Organization/Facility Name]**<br>Phone number:<br>Fax:<br>Email:<br>Address:<br>Hours:<br>Services offered:<br>Populations served:<br>Send referral letters by: [phone/email/hard copy/fax]

Appendix D: Referral Letter Template

Date:___________________________________ **Referral to:** _______________________________

To whom it may concern,

Kindly attend to the following client whose details are listed below:

Name:____

Address:_________________________________ **Telephone No.:**____________________________

____

Reason for referral:

____

____

____

____

____

____

Referred by

Name:___________________________________ **Signature:**_______________________________

Please do not hesitate to contact us at [insert organization name and add phone number] should you require further information. If your facility or program is unable to assist this client, please refer her back to our facility or a suitable alternate facility that will be able to assist her.
